# Medical Opinion and Movement

**Published:** 1909-01-23

**Authors:** 


### Medical Opinion and Movement.

The researches of Sir A. E. Wright into the coagulation time of the blood, the possibility of influencing it by various drugs, and the therapeutic effects of so doing, are among the earliest and best known of his investigations. The coagulometer which he invented has, with modifications, held the field for fifteen years as a simple and reasonably accurate apparatus; and upon its findings a large superstructure of observation and theory has been built up. Dr. Addis advances, in the *Quarterly Journal of Medicine*, some damaging criticisms both of this coagulometer and of Wright's researches into coagulation times and the influence of calcium salts and citric acid upon them. First of all he used the ingenious method described by Blair Bell (in the *British Medical Journal* of 1907) to ascertain whether the administration of these drugs by the mouth does lead to an alteration of the calcium content in the blood, or rather of the calcium content in ionic form, which can alone affect coagulation. He concludes that to give calcium salts does increase the ionic calcium of the blood, and that to give citric acid diminishes it. But he denies that these alterations affect the coagulation time of the blood, as tested by his own coagulometer, which he believes to be free from many of the errors he has found in Wright's. He finds that the coagulation time is quite unaffected by large or small doses of soluble lime-salts or of citric acid given by the mouth; and he thinks that this is due to the fact that the alterations in the percentage composition of the blood, which can be thus produced, are insufficient to bring about the results which Wright has claimed for calcium and citrate therapy.

---

The speed limit of the human heart is the subject of an interesting communication from Drs. Hertz and G. W. Goodhart, who have had under treatment in Guy's Hospital a woman in whom, for over a year, the auricles and ventricles seem to have been working in complete dissociation. Continuously for that space of time the auricles have contracted at a speed varying between 216 and 236 times per minute. The determinations are founded on jugular tracings controlled by $x$-ray observations of the right auricle and by inspection of the retinal veins, and there is no reason to suppose that they are not accurate. During the same period the ventricular systoles have been, during rest in bed, about 80 to the minute, though their number can be easily increased by exercise or atropine to 120 or more. The auricular contractions, *per contra*, are absolutely unaffected by drugs, posture, or work, and are most commonly 234 per minute, without variation during long periods. The authors consider from the clinical history of the patient, who is a woman of thirty-eight years of age, that she had an attack of endocarditis following scarlet fever in youth, and resulting in mitral stenosis; and, further, that the vagal terminations in the walls of the auricles must have been totally destroyed and all vagus control thus lost. They proceed to advance the interesting speculation that, for this patient at least, the speed limit of the human heart is 236 beats per minute, by which they mean the most rapid rate of contraction which can be kept up for long periods. They do not think that any rate in excess of this can be maintained more than temporarily, though allowance for individual variation must be made. They also criticise any record of cardiac contractions in excess of 200 per minute, unless proved by tracings.

---

A remarkable experiment in human vivisection, the consequences of which have been watched for a period measured by years, is recorded by Drs. Rivers and Head in *Brain*. In 1903 the latter physician, when in perfect health and condition, was, at his own request, operated upon by a surgeon, who removed under anæsthesia a small portion of the radial nerve on one side close to its origin from the musculo-spiral; the external cutaneous nerve was also divided, and both nerves were sutured with silk. This was done for the purpose of gaining accurate information upon the various kinds of sensibility, deep and superficial, or protopathic and epicritic, and was undertaken because of the difficulty of obtaining a long series of accurate observations in those who have been the subjects of injury. Also accidental injuries of cutaneous nerves alone without injury to muscular branches are excessively rare, and may be complicated by other factors such as alcoholism. The operation did not interfere with sensibility to the tactile and painful aspects of pressure; but the whole of the affected area became insensitive to prick, to heat, and to cold. Localisation of the compass points was preserved, but two points applied simultaneously were felt as one.

---

The cutaneous analgesia produced by the nerve lesion was first noticed to be distinctly diminishing on the hand forty-three days after the operation. Thirteen days later that on the forearm was markedly diminished, and the thumb had become sensitive to pricks. Eighty-six days after the section the whole forearm responded to pricks, and much of the hand. Cold was appreciated only at the tip of the thumb, and 50° C. gave rise to no sensation of heat. Restoration of function went on steadily and continuously, except for a short time during one winter; and five hundred and sixty-seven days after the operation only a small area of skin in the neighbourhood of the knuckles of the index and middle finger remained slightly insensitive. Even at the present time this part is in a purely protopathic condition; sensitive only to pricks, to cold and heat, which still cause diffused instead of localised sensations. The deductions which are drawn from this work are concerned largely with the functions of the afferent fibres of motor nerves. Localisation is in all probability the sum of two sets of sensations, one arising from deep, the other from cutaneous stimulation. Deep sensibility is responsible for our knowledge of the position of our limbs in space.

In a recent communication to the Académie de Médicine, Dr. Martin du Magny, of Bordeaux, draws attention to the "distant voice" sign as a means of diagnosing compression of the bronchial tubes. The patient should be in a sitting position and is requested to cough or repeat the number 333, or in the case of infants their cry is sufficient. At the same time auscultation is carried out by placing the stethoscope alternately at the apex and at the base of the chest behind. At the apex the voice and cough sound immediately under the ear; at the base they appear at a distance. The diminution in sound at the base is proportional to the compression. In the case of unilateral lesions a comparison of the sound at the two bases enables the physician to decide to which side the lesion belongs. In the case of bilateral lesions the side on which the compression is greater can be distinguished by the sound appearing more distant at that base. According to the author this sign affords a valuable means of diagnosis in cases of enlarged mediastinal glands. The sign is pathognomonic, constant, and can be distinguished in an early stage of the disease.

An interesting discussion took place at a recent meeting of the Societé de Médecine de Paris upon the value of the operation of prostatectomy. The surgeons were almost unanimous in acclaiming the highly satisfactory results obtained by Freyer's operation—that is the transvesical prostatectomy. M. Georges Luys, in summing up his views on the question may be said to voice the opinion which is generally held by surgeons at the present time. He contended that in regard to the indications for prostatectomy all patients who suffer from retention of urine should submit to operation, provided they are not cachectic and their renal functions are sufficient. Patients who have to submit to repeated catheterisation are always in danger, and some slight mistake can easily lead to the most severe complications. He maintained, therefore, that it is the duty of the surgeon to free the patient as soon as possible from the use of the catheter and to propose operation. With regard to the choice of operation the transvesical is much to be preferred to the older perineal method. The prostate can be removed in its entirety and there is no fear of recurrence of the trouble. Other surgeons similarly dilated on the constant possible attending dangers of catheter life and the desirability in consequence of advocating operation whenever the condition of the patient rendered surgical interference justifiable.

Dr. J. Babinski, the eminent neurologist, has contributed an important and highly interesting article to *La Semaine Médicale* on the subject of hysteria. The main theme of this contribution is that the traditional use of the term hysteria has led to much confusion in the diagnosis and treatment of neuropathic conditions, and he seeks to define clearly what morbid conditions fall properly under this heading, and what should be excluded. He points out in the first place three principal causes which have led to an excessive extension of the term hysteria beyond its proper application. In the first place, mistakes have been made in diagnosis by which organic affections have been interpreted as hysterical. This fact is readily recognised by all neurologists, and requires but little comment. Thanks to certain definite signs which have been discovered in recent years, and by which neuropathic conditions of organic origin can be detected and differentiated in the initial stages, such mistakes are much less frequent. In the second place hysteria has been confused with fraud and simulation. The author points out the difficulty of distinguishing these two conditions in many cases because the phenomena simulated may be identical with those produced by the subject of hysteria. The difference lies in the mental attitude, which in the one case is conscious and voluntary, in the other case unconscious or subconscious. So far, the author's arguments conform to usually accepted views and present no point of contention. It is under the third heading that he strikes out on a somewhat new line of thought. He contends that under hysteria different nervous conditions have been included which ought to be distinguished from one another. He differentiates three kinds of nervous phenomena which have received the appellation "hysterical," but which, he contends, are separate and distinct, and should not therefore be brought under one common denomination.

Those phenomena to which he would restrict the term "hysterical", or for which he proposes also an alternative epithet, are such as can be reproduced or arrested by suggestion. Such phenomena are convulsive fits; paralyses; contractures; tremors; choreic movements; disturbances of phonation, respiration and sensation; anæsthesia and hyperæsthesia. On the other hand, it is impossible by suggestion to exaggerate or abolish the tendon reflexes, to produce disturbances in the pupillary or cutaneous reflexes. Suggestion cannot produce vaso-motor, secretory or trophic disturbances, hæmorrhages, anuria, albuminuria or fever. All these phenomena therefore are to be excluded from the province of hysteria, the essential criterion being that they cannot be produced by suggestion, and do not fall, therefore, within the power of volition. The term which the author proposes instead of hysteria in this restricted sense is "pithiatism," from πειθώ persuade, and ιατος curable. From such "pithiatic" phenomena and conditions he would distinguish "emotive" and "reflex." He contends that the emotions do not play an important *rôle* in truly hysterical or "pithiatic" conditions; but, on the other hand, vaso-motor, secretory disturbances, and the like are not under the control of volition, and should be therefore clearly separated. For the same reason he makes this third "reflex" group of functional disturbances. It is impossible to reproduce all the arguments he develops in support of these distinctions, but his main purport may be indicated in the guidance they afford to the physician in his diagnosis and treatment of these conditions. With a clear and precise conception of such neuropathic conditions as are within the volitional control of the patient, he is able to recognise and distinguish what is suitable for "pithiatic" treatment, and he can set to work with the full knowledge that by placing the patient in suitable surroundings, by personal influence and well-measured psycho-therapeutic methods the morbid condition can be cured.

---

The sterilisation of the skin, preliminary to operation, by tincture of iodine is advocated by Dr. Antonio Grossich, of Fiume, in the *Centralblatt für Chirurgie*. The suture line is also painted with the same solution by this surgeon, with excellent results. The pre-operative cleansing includes no washing or scrubbing with soap and water, as this is believed to swell up the scales of the epidermis, and so prevent their due penetration by iodine. The patient takes a bath the day before operation, and next morning the whole field is freely painted with an alcoholic solution of iodine of 15 per cent. strength. After the patient is anæsthetised, another coat is added before the incision is made. Hairy parts should be shaved, as in other methods of preparation; but if this cannot be carried out several hours beforehand it is recommended that dry shaving should be adopted. For emergency operations such as strangulated hernia, for example, this method is evidently a great saving of time: it is said by those who use it to be very effective, and quite free from ill effects, even when one third of the cutaneous surface has been thus treated. Other surgeons in this country who have tried the plan commend it highly, though they do not all omit the usual routine of soap and water, turpentine, ether, and so on.

---

Yet another of the vegetable panaceas advocated for cancer has been found, on independent investigation, not to bear out the claims made for it. Alnus glutinosa is the remedy, which has been under observation at the Cancer Hospital in this respect, with a view to corroboration of the experience which led Dr. Grey to recommend it. The result of the trial is a complete negation of any curative or palliative properties alleged to exist in this drug, which was fully investigated in nine cases. Seven of these were carcinomata of the breast, wherein opportunities for observing local changes in the growth itself, over and above the constitutional, were afforded. In every case the disease pursued its natural course. The local tumours increased in size and broke down into ulcers. Lymphatic dissemination went on unchecked, and pain was equally unaffected. The patients went steadily downhill, and several of them are already dead. The three members of the staff who undertook the inquiry are all satisfied that the treatment has in their hands been followed by no beneficial results, and consider it established that alnus glutinosa has no specific action upon cancerous processes. Disappointing as a blank result must always be in connection with novel forms of treatment of cancer, the truth, the whole truth, and nothing but the truth is absolutely essential to scientific progress; and to record every scrap of evidence, even when it is thus negative, is essential, so that others may be saved from wasting their time in fruitless labour.

Diffuse septic peritonitis due to appendicitis is the subject of a paper by Dr. R. H. Fowler, who analyses 69 such cases treated in hospital in ten years. These are dealt with very thoroughly; the modifications of procedure, after treatment, and results are set out for each successive year, particularly with regard to the semi-erect position advocated by Dr. G. R. Fowler and generally known by his name. The main facts as to mortality are that sixteen patients out of thirty-two thus treated died, whereas eighteen out of twenty-two not propped up did so. The total mortality of the whole series works out at 69.5 per cent. Stress is laid upon early diagnosis and operation as a more important factor in improving these results than any modification in technique can be. Equally is early institution of postural drainage, both before laparotomy and after, insisted on. The technique of operation recommended is a small incision over McBurney's point, rapid but gentle treatment of the primary focus; avoidance of evisceration; peritoneal lavage; and free drainage, especially by posterior colpotomy in women. It is expressly stated that no cases have been included which were merely large abscesses or inflammations confined to one quadrant or to one half of the abdomen. The results obtained are not perhaps equal to the optimistic forecasts of some of the most enthusiastic advocates of Fowler's position; but they are encouraging, and are stated with a most admirable candour.

---

Extroversion of the bladder is a condition which, while fortunately rare, is very difficult to treat successfully. In the *Edinburgh Medical Journal* are reported two cases operated upon respectively by Mr. A. Thomson and Mr. J. W. Dowden. The former adopted the method associated with the name of Peters, and implanted both ureters, after separation from the bladder, into the rectum. The immediate result was satisfactory in the extreme; for while at first the urine was passed in quantities of an ounce at a time, and there was nocturnal incontinence, within three weeks the rectum would tolerate eight to ten ounces, and the nocturnal difficulty was got over. For more than a year matters went well, and the exposed vesical mucosa gradually shrank until it was no larger than a five shilling piece; but incontinence at night was not infrequent. Gradually however, symptoms of renal disease supervened, almost certainly due to ascending infection, and death from uræmia ensued two years and three months after the operation. In the second case the surgeon excluded a loop of colon from alvine contamination by performing a lateral anastomosis between the iliac and pelvic parts of the large gut. The ureters with the vesical mucous membrane between them were then implanted into the excluded loop; the idea being to avoid ascending pyelitis and nephritis. The rest of the bladder mucosa was removed. Shock was marked, but a good recovery was made. Three months after the operation Mr. Dowden reports that the urine is sometimes retained for two hours; but he thinks that renal mischief has already commenced.

